# 高原地区初诊急性白血病临床特征及治疗情况

**DOI:** 10.3760/cma.j.cn121090-20240514-00182

**Published:** 2024-12

**Authors:** 秋燕 何, 安丽 赖, 澳 张, 玲娜 汪, 格桑曲珍, 平措卓嘎, 次仁, 秀明 陈, 益西拉姆, 少伟 邱, 辉 魏, 建祥 王, 白玛央金, 广吉 张

**Affiliations:** 1 西藏自治区人民医院，拉萨 850000 People's Hospital of Tibet Autonomous Region, Lhasa 850000; 2 中国医学科学院血液病医院（中国医学科学院血液学研究所），血液与健康全国重点实验室，国家血液系统疾病临床医学研究中心，细胞生态海河实验室，天津 300020 State Key Laboratory of Experimental Hematology, National Clinical Research Center for Blood Diseases, Haihe Laboratory of Cell Ecosystem, Institute of Hematology & Blood Diseases Hospital, Chinese Academy of Medical Sciences & Peking Union Medical College, Tianjin 300020, China

**Keywords:** 白血病，淋巴细胞，急性, 白血病，髓系，急性, 高原, 疗效, Leukemia, lymphoblastic, acute, Leukemia, myeloid, acute, Plateau, Prognosis

## Abstract

**目的:**

描述及分析高原地区急性白血病患者的临床特征及疗效。

**方法:**

回顾性统计分析2010年2月至2023年4月西藏自治区人民医院收治的急性白血病患者的临床资料，包括血常规、细胞形态学、流式细胞术免疫分型、分子生物学及细胞遗传学等，对患者进行随访，并进行生存分析。

**结果:**

研究共纳入105例急性白血病患者，包括急性淋巴细胞白血病（ALL）24例、急性髓系白血病（AML）62例，无系别资料的急性白血病患者19例。11例具备流式细胞术免疫分型资料的ALL患者均为B-ALL。57例AML患者具有分型资料FAB亚型以M_2_为主（25例），其次为急性早幼粒细胞白血病12例，M_5_ 6例，M_4EO_ 5例，M_1_ 4例，M_4_ 4例，M_0_ 1例。经1个疗程诱导化疗后可评价疗效者48例，其中ALL 14例，APL 6例，AML（非APL）28例，完全缓解（CR）率分别为57.1％（8/14）、100％（6/6）、53.6％（15/28）。中位随访37（95％*CI* 17～57）个月，ALL患者的中位无事件生存（EFS）期为2（95％*CI* 0～9）个月，中位总生存（OS）期为3（95％*CI* 0～9）个月；APL患者中位EFS期及OS期未达到；核心结合因子相关AML（CBF-AML）患者的中位EFS期及OS期分别为10（95％*CI* 0～21）个月、13（95％*CI* 3～23）个月，非CBF-AML（包括无染色体核型及融合基因资料患者）患者的中位EFS期及OS期均为2（95％*CI* 1～3）个月，不同亚型AML患者的EFS期及OS期差异有统计学意义（*P* 值均<0.01）。按收治年份将ALL患者分成2010–2019年和2020–2023年两组，后者较前者EFS（10个月对1个月，*P*＝0.16）及OS（15个月对1个月，*P*＝0.10）呈改善趋势；按收治年份将AML患者分成2010–2015年和2016–2023年两组，后者较前者EFS（10个月对3个月，*P*＝0.27）及OS（12个月对3个月，*P*＝0.12）呈改善趋势。

**结论:**

高原地区急性白血病疗效虽然较国内发达地区仍有差距，但近年来已取得了较大进展，推进急性白血病精准诊断和治疗有利于改善患者预后。

## Abstract (en)

**Objective:**

This study aimed to retrospectively analyze the clinical characteristics and prognosis of patients with acute leukemia in the plateau.

**Methods:**

The clinical information of patients diagnosed with acute leukemia from February 2010 to April 2023 at the People's Hospital of Tibet Autonomous Region was reviewed and collected, including blood cell count, morphology, immunophenotype, cytogenetics, and molecular data. Survival analysis was conducted to analyze the outcome of patients with acute leukemia.

**Results:**

This study enrolled 105 patients with acute leukemia, including 24 with acute lymphoblastic leukemia（ALL）, 62 with acute myeloid leukemia（AML）, and 19 with acute leukemia without baseline data. Of the patients with ALL, 11 underwent bone marrow testing for immunophenotype, all of whom were B-cell lineage. The main FAB subtype of patients with AML was M_2_（25/57）, followed by M_3_（12/57）, M_5_（6/57）, M_4EO_（5/57）, M_1_（4/57）, M_4_（4/57）, and M_0_（1/57）. The complete remission rates of patients with ALL, acute promyelocytic leukemia（APL）, and AML（non-APL）after one course of induction therapy were 57.1％（8/14）, 100％（6/6）, and 53.6％（15/28）, respectively. The median event-free survival（EFS）and overall survival（OS）for patients with ALL were 2（95％ *CI* 0–9）and 3（95％ *CI* 0–9）months, respectively, with a median followup of 37（95％ *CI* 17–57）months. Patients with APL did not reach median EFS or OS, whereas the median EFS and OS for core binding factor AML（CBF-AML）cases were 10（95％ *CI* 0–21）months and 13（95％ *CI* 3–23）months, respectively, and patients with non-CBF-AML had inferior median EFS（2 months, 95％ *CI* 1–3）and OS（2 months, 95％ *CI* 1–3）（*P*<0.01）. Patients with ALL treated from 2020 to 2023 demonstrated trends toward better EFS（*P*＝0.16）and OS（*P*＝0.10）than those treated from 2010 to 2019. Similarly, trends toward superior EFS（*P*＝0.27）and OS（*P*＝0.12）were observed in patients with AML treated from 2016 to 2023, in comparison with those treated from 2010 to 2015.

**Conclusion:**

Progress in the treatment and prognosis of patients with acute leukemia in the plateau has been observed in recent years, which can be further promoted by precision diagnosis and tailored regimens.

急性白血病是造血干/祖细胞在不同分化阶段发生分化阻滞、凋亡障碍、恶性增殖导致的异质性血液系统恶性疾病，自然病程仅数月。虽然85％～90％的成人急性淋巴细胞白血病（ALL）和60％～70％成人急性髓系白血病（AML）在诱导化疗后可获得完全缓解（CR），大部分患者仍会复发，5年生存率仅30％～40％，严重威胁人类健康[1]–[2]。高原地区气候特殊、白血病诊疗起步晚，且文化传统一定程度上影响了白血病诊疗工作的开展，高原地区白血病诊治水平仍落后于内地。随着国家加大对口支援西藏的力度，医疗“组团式”援藏工作的开展[3]，高原地区白血病治疗发展迅速。目前仍缺乏对高原地区急性白血病患者临床特征、疗效情况的分析研究，因此，本研究通过回顾性分析高原地区白血病患者的临床资料及生存情况，旨在提高对该地区急性白血病的认识及医疗服务水平。

## 病例与方法

1. 病例：纳入2010年2月至2023年4月就诊于西藏自治区人民医院血液科的105例确诊急性白血病患者（WHO 2016标准），回顾性收集患者血常规、细胞形态学、流式细胞术免疫分型、分子生物学及细胞遗传学检查资料。

2. 随访：通过查阅患者临床资料及电话随访，随访截止时间为2003年5月26日。总生存（OS）期为初次诊断至任何原因导致的死亡或者删失的时间；无事件生存（EFS）期为初次诊断至诱导治疗失败、形态学复发或任何原因导致的死亡、删失的时间。

3. 不良反应评价：第1疗程诱导治疗不良反应中血液学毒性及非血液学毒性根据CTCAE 5.0标准进行评估。

4. 统计学处理：应用R 4.3.1进行统计学处理。分类变量以例数（百分率）描述，连续变量以中位数（范围）描述，组间分类变量比较采用卡方检验或Fisher精确概率法。对随访时间大于1个月的ALL、AML患者采用Kaplan-Meier法进行生存分析，并采用Log-rank检验进行组间比较。双侧*P*值<0.05为差异具有统计学意义。

## 结果

1. 患者基本资料：105例急性白血病患者的临床特征见表1。其中，藏族患者102例，汉族患者2例，门巴族患者1例。ALL 24例，AML 62例，无系别诊断资料的急性白血病19例；中位年龄分别为33（16～73）岁、37（14～78）岁、46（15～73）岁；居住地中位海拔高度分别为3 830（2 340～4 700）m、3 800（800～4 700）m、3 820（2 340～4 700）m；中位发病至就医时间分别为30（14～180）d、20（4～150）d、20（5～120）d；中位就医至诊断时间分别为5（2～6）d、5（1～6）d、2（1～5）d。AML中，57例患者有分型资料，FAB分型以M_2_为主（25例，43.9％），其次为急性早幼粒细胞白血病（APL）12例（21.0％），M_5_ 6例（10.5％），M_4EO_ 5例（8.8％），M_1_ 4例（7.0％），M_4_ 4例（7.0％），M_0_ 1例（1.8％）。ALL中，11例患者具有流式细胞术免疫分型资料，均为B-ALL。具有染色体核型或融合基因资料的患者中，ALL伴t（9;22）（q34;q11）/BCR∷ABL1 9例（69.2％），ALL伴KMT2A重排1例（8.3％）；AML伴t（15;17）（q24;q21）/PML∷RARA 7例（19.4％），AML伴t（8;21）（q22;q22）/RUNX1∷RUNX1T1 8例（22.2％），AML伴inv（16）（p13q22）/CBFB∷MYH11 5例（13.9％），AML伴BCR∷ABL1 1例（2.8％）。

**表1:** 105例高原地区急性白血病患者的临床特征

临床特征	ALL（24例）	AML（62例）	无系别资料急性白血病（19例）
性别［例（％）］			
男	8（33.3）	39（62.9）	12（63.2）
女	16（66.7）	23（37.1）	7（36.8）
年龄［岁，*M*（范围）］	33（16～73）	37（14～78）	46（15～73）
民族［例（％）］			
藏族	24（100.0）	60（96.8）	18（94.7）
汉族	0（0）	1（1.6）	1（5.3）
门巴族	0（0）	1（1.6）	0（0）
海拔高度［m，*M*（范围）］	3 830（2 340～4 700）	3 800（800～4 700）	3 820（2 340～4 700）
发病至就医时间［d，*M*（范围）］	30（14～180）	20（4～150）	20（5～120）
就医至诊断时间［d，*M*（范围）］	5（2～6）	5（1～6）	2（1～5）
WBC［×10^9^/L，*M*（范围）］	68.5（1.0～649.0）	7.6（0.4～237.0）	31.6（0.7～314.0）
HGB［g/L，*M*（范围）］	85（33～134）	73（25～161）	55（26～116）
PLT［×10^9^/L，*M*（范围）］	20.0（2.0～258.0）	17.5（2.0～580.0）	14.0（2.0～132.0）
乳酸脱氢酶［U/L，*M*（范围）］	448（107～1 050）	346（127～2 270）	533（214～3 760）
骨髓原始细胞［％，*M*（范围）］	81.0（56.0～96.0）	58.2（10.0～98.0）	63.5（34.5～92.5）
外周血原始细胞［％，*M*（范围）］	88.0（28.0～98.0）	82.5（18.0～98.0）	87.5（32.0～100.0）
FAB分型［例（％）］^a^			
M_0_	NA	1/57（1.8）	NA
M_1_	NA	4/57（7.0）	NA
M_2_	NA	25/57（43.9）	NA
M_3_	NA	12/57（21.0）	NA
M_4_	NA	4/57（7.0）	NA
M_4EO_	NA	5/57（8.8）	NA
M_5_	NA	6/57（1.5）	NA
正常核型［例（％）］^a^	1/6（16.7）	7/24（29.2）	NA
复杂核型［例（％）］^a^	1/6（16.7）	4/24（16.7）	NA
t（15;17）/PML∷RARA［例（％）］^a^	NA	7/36（19.4）	NA
t（8;21）/RUNX1∷RUNX1T1［例（％）］^a^	NA	8/36（22.2）	NA
inv（16）/CBFB∷MYH11［例（％）］^a^	NA	5/36（13.9）	NA
t（9;22）/BCR∷ABL1［例（％）］^a^	9/13（69.2）	1/36（2.8）	NA
KMT2A重排［例（％）］^a^	1/12（8.3）	0（0）	NA
CR［例（％）］^a^	8/14（57.1）	APL: 6/6（100） 非APL: 15/28（53.6）	0/1（0）

**注** ALL：急性淋巴细胞白血病；AML：急性髓系白血病；APL：急性早幼粒细胞白血病；CR：完全缓解，NA：不适用。^a^数据为阳性例数/检测例数

2. 患者治疗方案、治疗反应率及耐受性：15例ALL患者接受了诱导化疗，Ph^+^-ALL中，多药化疗+酪氨酸激酶抑制剂（TKI）诱导化疗7例，TKI+糖皮质激素诱导化疗1例；另7例无核型资料ALL患者采用VP方案（长春碱类+糖皮质激素）为基础的诱导化疗，其中含门冬酰胺酶方案3例。诱导后CR率57.1％（8/14），接受巩固治疗1例。7例患者有诱导治疗不良反应资料，6例发生Ⅲ～Ⅳ级中性粒细胞减少，6例发生Ⅲ～Ⅳ级血小板减少，6例发生Ⅲ级血红蛋白减少；1例出现Ⅲ级恶心及呕吐，2例出现Ⅲ级肺感染，Ⅲ级转氨酶升高2例，Ⅲ级胆红素升高1例。

6例APL患者接受了诱导治疗，其中4例高危患者中，采用全反式维甲酸（ATRA）+亚砷酸+化疗2例，ATRA+亚砷酸2例；2例低（中）危患者中，采用ATRA+复方黄黛片1例，ATRA+柔红霉素1例。6例患者均获得CR，CR后4例患者采用ATRA+复方黄黛片巩固治疗，1例患者采用3个周期DA方案（柔红霉素+阿糖胞苷）后接受ATRA+复方黄黛片巩固治疗，余1例患者使用ATRA巩固治疗。1例患者有诱导治疗不良反应资料，该患者发生Ⅳ级中性粒细胞减少、Ⅳ级血小板减少、Ⅲ级血红蛋白减少、Ⅲ级肺感染。

30例AML（非APL）患者接受了诱导化疗，采用“3+7”强化疗方案诱导化疗18例，其中DA方案16例，IA方案（伊达比星+阿糖胞苷）1例，EA方案（依托泊苷+阿糖胞苷）1例，CR率为61.1％（11/18）；采用低强度方案诱导化疗12例，其中阿扎胞苷或地西他滨联合小剂量化疗6例，阿扎胞苷联合维奈克拉1例，阿扎胞苷单药1例，CAG方案（阿克拉霉素+阿糖胞苷+G-CSF）2例，DAG方案（柔红霉素+阿糖胞苷+G-CSF）1例，HAG（高三尖杉酯碱+阿糖胞苷+G-CSF）方案1例，CR率为40.0％（4/10）（表1）。CR后接受巩固治疗14例，巩固化疗方案包括中大剂量阿糖胞苷（6例）、DA方案（5例）、EA方案（1例）、AA方案（阿柔比星+阿糖胞苷，1例）、阿扎胞苷（1例）。21例患者有诱导治疗不良反应资料，18例患者发生Ⅲ～Ⅳ级中性粒细胞减少，20例患者发生Ⅳ级血小板减少，21例患者均发生Ⅲ～Ⅳ级血红蛋白减少；11例患者出现Ⅲ级肺感染。

3. 不同年份患者接受治疗率及治疗反应率：2010–2015年、2016–2021年、2022–2023年，分别收治急性白血病患者23、80、11例，接受诱导治疗的患者分别为4（17.4％）、41（51.3％）、7（63.6％）例，接受诱导治疗的患者比例呈上升趋势。诱导治疗1个疗程CR率分别为25.0％、59.0％、83.4％，不同年份CR率差异虽无统计学意义（*P*＝0.68），但仍呈上升趋势。相比2010–2015年无患者在CR后继续接受巩固治疗，2016–2021年及2022–2023年患者获得CR后继续接受巩固治疗的例数分别为14、2例（*P*＝0.46）（表2）。

**表2:** 不同年份高原地区急性白血病患者接受治疗率及治疗反应率

年份	例数	诱导化疗例数	治疗率（％）	1个疗程诱导化疗CR^a^	CR后巩固治疗例数	CR后巩固治疗率（％）
2010–2015	23	4	17.4	1/4（25.0）	0	0
2016–2021	80	41	51.3	23/39（59.0）	14	60.9
2022–2023	11	7	63.6	5/6（83.3）	2	40.0

*P*值			0.11	0.68		0.46

**注** CR：完全缓解；^a^数据为CR例数/可评估疗效例数（％）

4. 患者生存情况：对53例随访时间大于1个月的ALL和AML患者进行生存分析，中位随访时间为37（95％*CI* 17～57）个月。15例ALL患者的中位EFS期为2（95％*CI* 0～9）个月，中位OS期为3（95％*CI* 0～9）个月（图1A）。截至末次随访，4例ALL存活，其中1例存活超过5年。38例AML患者中位EFS期为7（95％*CI* 2～12）个月，中位OS期为8（95％*CI* 4～16）个月；其中APL患者中位EFS期及OS期均未达到，核心结合因子相关AML（CBF-AML）患者中位EFS期及OS期分别为10（95％*CI* 0～21）个月、13（95％*CI* 3～23）个月，非CBF-AML（包括无染色体核型或融合基因资料患者）患者中位EFS期及OS期均为2（95％*CI* 1～3）个月，不同亚型AML患者的EFS及OS差异有统计学意义（*P*<0.01）（图1B）。截至末次随访，10例AML存活，包括8例APL患者2例CBF-AML患者。

**图1:**
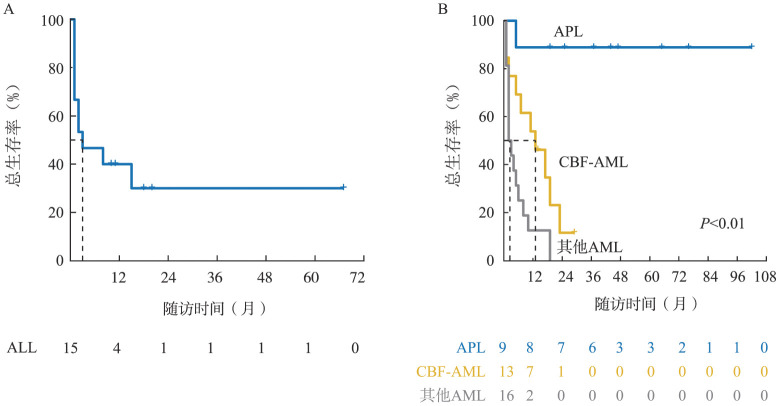
高原地区急性白血病患者生存曲线 **A** 急性淋巴细胞白血病（ALL）患者总体生存曲线；**B** 急性髓系白血病（AML）患者总体生存曲线 **注** APL：急性早幼粒细胞白血病；CBF：核心结合因子

按本院收治年份将ALL患者分为两组：2020–2023年组9例，2010–2019年组6例；2020–2023年组中位EFS期（10个月，95％*CI* 4～16）较2010–2019年组（未达到）呈延长趋势（*P*＝0.16），前者中位OS期（15个月，95％*CI* 1～29）较后者（未达到）亦呈改善趋势（*P*＝0.10）。按本院收治年份将AML患者分为两组：2010–2015年组6例，2016–2023年组32例；随访发现，2016–2023年组中位EFS期（10个月，95％*CI* 4～15）较2010–2015年组（2个月，95％*CI* 0～6）呈延长趋势（*P*＝0.27）,前者（11个月，95％*CI* 0～23）较后者（2个月，95％*CI* 0～6）的中位OS亦呈改善趋势（*P*＝0.12）。

## 讨论

随着强烈化疗、靶向治疗、免疫治疗及异基因造血干细胞移植的应用及改进，急性白血病患者生存得到极大改善，部分患者甚至能获得治愈[4]–[7]。本研究是国内首次分析高原地区急性白血病患者临床特征及疗效的报道，为认识该地区急性白血病诊疗现况提供可靠数据。本研究纳入的患者均居住于高原地区，ALL及AML患者的初发中位年龄与国内报道一致[8]–[9]。在援藏工作的大力支持下，高原地区已改变以往对急性白血病仅作形态学分型诊断的情况，依托第三方实验室逐步开展免疫、细胞遗传学及分子生物学检查，可对急性白血病患者做出精准诊断，有利于指导下一步个体化治疗[10]。对实施了染色体核型及相关融合基因检查的患者进行统计发现，AML伴t（15;17）（q24;q21）/PML∷RARA、AML伴t（8;21）（q22;q22）/RUNX1∷RUNX1T1、AML伴inv（16）（p13q22）/CBFB∷MYH11、AML伴BCR∷ABL1的比例与国外文献报道存在差异[11]–[12]，分析原因，不排除人种差异、具备染色体核型和融合基因等检查结果的患者例数过少的可能，需扩大样本量进一步研究高原地区急性白血病各分子亚型的占比。

相比儿童ALL超过90％的治愈率，成人ALL患者由于更易携带高危细胞、分子遗传学异常，如Ph染色体，长期生存的患者比例远低于儿童ALL患者[1],[13]。TKI联合化疗的方案极大改善了Ph^+^-ALL患者的预后，争取早期深度缓解有利于此类患者的长期生存，甚至不需异基因造血干细胞移植[14]–[15]。本研究中，大部分Ph^+^-ALL患者采用了TKI联合化疗的方案，但由于巩固治疗率低，患者预后不佳。此外，多项研究证实，采用强化门冬酰胺酶的儿童样方案，可显著改善成人ALL患者的预后[16]–[17]。本研究中，3例ALL患者采用了含门冬酰胺酶的儿童样方案，其中1例患者长期存活，亦提示含门冬酰胺酶的儿童样方案是有效治疗方案。

APL经ATRA联合砷剂治疗已成为可治愈的疾病，近年，多项研究证实ATRA联合口服砷剂即复方黄黛片治疗中低危APL的有效性，使APL的口服家庭治疗成为可能[18]–[19]。中低危APL口服家庭治疗更有利于在高原地区开展及推广，可改善APL患者的预后，减少住院率及缩短住院时长，极大减轻患者疾病负担[20]。本研究中1例低危APL患者诱导及巩固治疗采用了ATRA联合复方黄黛片，截至末次随访仍存活，提示APL口服家庭治疗的有效性，值得进一步推广。虽然可评价疗效的APL患者例数较少，截至末次随访，9例APL患者中仅1例死亡，5年OS率接近90％，生存率与国内外研究相当[18]。

CBF-AML包括AML伴t（8;21）（q22;q22）/RUNX1∷RUNX1T1、AML伴inv（16）（p13q22）或t（16;16）（p13;q22）/CBFB∷MYH11，多项研究证实CBF-AML患者预后良好，3年存活率在60％以上[5],[11],[21]。本研究中，CBF-AML较非CBF-AML患者生存呈上升趋势，但长期存活率低，分析原因，主要与部分患者CR后未继续接受巩固治疗相关，提示促进患者接受足够疗程化疗的重要性。

生存分析表明，高原地区急性白血病患者疗效与国内外队列研究报道的结果仍存在较大差距[11],[21]。分析原因，可能与部分患者携带不良细胞遗传学异常有关；此外，既往患者治疗率低，且部分患者未能接受标准剂量和足够疗程的化疗，早期死亡率高，长期存活患者比例较低，也是生存较差的重要原因；因地区广、病例经济水平差异较大，研究具有一定选择偏倚。近年来，随着国家加大对口援藏力度，急性白血病患者治疗率呈上升趋势，患者生存率呈改善趋势。因此，促进患者接受足够疗程的治疗及加大个体化治疗的推广将有利于进一步改善高原地区急性白血病患者的生存。

综上所述，高原地区急性白血病疗效虽与国内发达地区及国外存在差距，但较前已取得了较大进展，对该地区急性白血病患者展开个体化治疗，有利于减轻患者疾病负担，改善预后。
